# Victoria Hospital for Children

**Published:** 1901-11-30

**Authors:** 


### VICTORIA HOSPITAL FOR CHILDREN.

The Banqueting Hall of the Hotel Cecil was filled with a distinguished gathering on the 20th instant, on the occasion of the festival dinner in aid of the building fund of the Victoria Hospital for Children, Chelsea. The Commander-in-Chief, Field Marshal Lord Roberts, took the chair, and he was supported by the President of the Hospital, his Excellency Lord Cadogan, and a brilliant company of friends of the institution. After the customary loyal toasts had been duly honoured, Sir Charles Dilke in an able and erudite speech proposed the health of "The Imperial Forces." This was responded to by Admiral Sir Arthur Farquhar and Major-General Webber, and drunk with enthusiasm.

Lord Roberts then proposed "Success to the Victoria Hospital." He said that when Lord Cadogan did him the honour of asking him to preside at that dinner he felt some misgivings as to the propriety of acceding to the request, since he thought there were many gentlemen who could much more appropriately fill the chair and tell from personal knowledge all about the Victoria Hospital, and the urgent need there was for its extension. But that request was made more than two years ago, when he was serving in Ireland, with Lord Cadogan as Lord Lieutenant, and therefore his commanding officer, and in the interests of discipline he felt it would be improper to refuse the request. The dinner, for one reason and another, had been a long time coming off, but it was in fulfilment of that promise he was there that night. Having undertaken the task, he thought the best thing he could do was to visit the Victoria Hospital and judge for himself how matters stood. He had read the report of the meeting at Grosvenor House in 1898, when the necessities of the situation were eloquently set forth by the speakers there; but his visit to the hospital had convinced him that the case had really been under- rather than over-stated, and that the defects of the present hospital made a new one absolutely necessary. From the first annual report, in 1868, it appeared that the institution was intended mainly for out-patients or for only a very limited number of in-patients, since there were then only six beds. A little later this was increased to 24, but during the first twelve months of the hospital's existence there were only 39 in-patients, while the out-patients numbered over 5,000. As it became more known the committee extended the 24 beds to 70, but this was more than the superficial and cubical area of the rooms would properly permit, with the result that there were three epidemics of diphtheria; and at last, on the representation of the sanitary authorities, the 70 beds were reduced to 42, and even for these the accommodation was most inadequate. Last year the number of in-patients was 560, while the out-patients were close on 50,000. Statistics showed that there were in Chelsea some 260,000 children under 15, with only 124 hospital beds available for them; and they were met that evening to support a well-considered project for remedying, to some extent, this deplorable state of things on the vacant site beside the present building. There was fortunately room to erect a hospital to contain 96 beds, with a separate ward for infectious cases. This was very necessary, because now if an infectious case occurred they had to give up the use of a whole ward. The estimated cost of the new hospital was £30,000, of which only about £6,000 had so far been promised. Having explained the facts, he did not think it necessary to enlarge on the need for a new and commodious hospital, and he hoped the appeal now made would be liberally responded to, and that there would be provided the funds for the erection of a building worthy of the object and of the neighbourhood. Loud cheers greeted this speech, to which Earl Cadogan and Sir William Broadbent replied.

Lord Cadogan thought the very best he or any of them could do would be to say "ditto to Lord Roberts." He thanked him heartily for the businesslike and forcible way he had put their case before the public. He thought they had reason to complain that the charities of the wealthier parts of London did not meet with the support they should. He was well aware that many of those who had residences elsewhere and only spent a short three months of the year in town were loth to support institutions in the south-western and western districts, considering that they had responsibilities in other places and that the residents in those localities should be left to manage their own affairs. But it was forgotten how round the great houses and flats of the south-west there were poorer districts and even slums where the conditions of life were almost a scandal. The Victoria was the only large children's hospital for the neighbourhood, and now that the Belgrave Hospital had been moved to the other side of the river they were in the position of having to undertake the added work. He was glad to announce that he had a few hours before received a telegram from the Duke of Westminster with a liberal donation, and he could wish him no greater happiness in life than to follow in the footsteps of the late Duke in such matters. To them all he would say that he felt sure they would never have need to regret the generosity to which he hoped they would be moved, and he hoped that outside that room the speech of the chairman would give the hospital the advertisement it so badly needed.

Sir William Broadbent laid stress on the fact that, while in a general hospital they often had the privilege of restoring the breadwinner to his work, in a children's hospital they were able to take a child who, but for that, would drag out a miserable existence, and send him out fitted to be an efficient workman, and to do his duty in his day and generation.

Mr. H. G. Everett, the Secretary of the hospital, then read the lists of subscriptions, which totalled £7,475, and £167 of new annual subscriptions.

Mr. A. C. Whitmore, M.P., then proposed the toast of "The Committee of Management and Medical Staff," whom, influenced by the prevailing military tone, he characterised as the War Office and the commanding officers in the field, so far as the Victoria Hospital for Children was concerned. He congratulated the management on the fact that the institution was popular in its own neighbourhood among those who benefited by its presence there, and that no whisper of hostile criticism on the management had ever been heard.

Mr. Alfred Farquhar, the Treasurer, said that it was owing to the absence, through the imperative instructions of his doctor, of their Chairman, Mr. Martin Smith, to whom he paid a warm tribute, that he was replying to the toast. He would, however, put the case before them as briefly as possible. Emphatically, then, the Victoria Hospital could resist no longer the influx of time and tide; it was tumbling down and had got to be rebuilt. It was not built originally as a hospital at all; it was only with the greatest difficulty they had been able to adapt it to their requirements, and a change could be delayed no longer. In face of the difficulties which confronted them they could allow no further time to elapse before commencing the work of reconstruction. They had happily the necessary ground at their disposal; the funds were lacking. The minimum required would be from £25,000 to £30,000, and towards that they had £6,000, and promises of £5 000 or £6,000 more. They might say they had nearly half the sum required within their reach and could feel they were half-way along their road. But the future was chaotic and uncertain, and a single glance at their most depleted balance-sheet would show how sore was their need. True, they were situated in the wealthy West End, but they did not receive the support that was due to such a priceless institution. Making all allowance for other claims, nothing could constitute a greater appeal than the condition of the unfortunate victims of poverty, of hereditary disease, and of the brutality or callousness of unnatural parents. Last year their income was some £5,200, as against an inevitable expenditure of over £6,000, leaving a deficit of £800, which was still represented by an undischarged loan from the bank of £500. Then there was their Convalescent Home at Broadstairs, by which they were enabled to send back to their parents hosts of little sufferers invigorated by their stay at the seaside. At times they had assistance from legacies, but of these last year the total was almost nil; and this year, again, all organisers of charitable funds had to admit that contributions had fallen off owing to the war funds. As regarded the working staff, since last they met in that way there had been one very sad change. They had lost Captain Blount, who for over 20 years had been a most devoted servant to the institution. They had now as Secretary Mr. Everett, by whose energy and devotion the interests of the hospital were being most thoroughly safeguarded. The speaker concluded with a tribute to the medical staff, and, after a reply from Mr. D'Arcy Power, the proceedings terminated.

